# Geographic Variation in Racial Disparities in Age-Adjusted Mortality Rates in Mississippi

**DOI:** 10.1007/s40615-024-02276-7

**Published:** 2025-01-20

**Authors:** Isaac M. E. Dodd, Yousaf Zafar, Malachi E. Scott, Richard F. Gillum

**Affiliations:** 1 Department of Medicine, Division of Hospital Medicine, University of Mississippi Medical Center, Jackson, MS USA; 2 Department of Medicine, Howard University College of Medicine, Washington, DC USA

**Keywords:** Mississippi, Healthcare disparities, Racial disparities, Age-adjusted mortality rates, County-level analysis, Geographic variation

## Abstract

**Background:**

Racial disparities in mortality rates have been well-documented in the last century. Intersectionality theory has helped to identify the root causes of these health disparities. Few studies have examined disparities using the latest data for the state of Mississippi.

**Methods:**

Mortality data for the state of Mississippi (MS) were obtained from the vital statistics program of the Centers for Disease Control & Prevention for years 1999–2020. The age-adjusted mortality rate (AAMR) for ages 35–84 years was calculated by county, gender—male (M) vs female (F), and race—black (B) vs white (W), among non-Hispanics (NH) for all causes of death.

**Results:**

In 2020, MS had the highest AAMR per 100,000 among states in the US: 1624.76 (1605.61–1643.91) for age group 35–84. In 1999–2020 combined, AAMR varied among counties for each gender-race group. High AAMR was concentrated in the delta region for NH black males (NHBM) and females (NHBF). This was less so for NH white males (NHWM) and not so for females (NHWF). The Black/White AAMR ratio among males and females was highest (1.42, 1.36) in the small metropolitan areas and lowest (1.18, 1.05) in the large fringe metropolitan areas. In 1999–2020 for NH males, the ratio of AAMR in NHB to NHW varied from 0.9 to 1.8. In NH females, the ratio varied from 0.9 to 2.2. In both genders, the ratio was significantly correlated with the percent of the population that was NH black.

**Conclusions:**

The AAMR in MS varied greatly among counties as did the Black/White ratio of AAMR. Further research is needed to explain this geographic variation in racial disparity.

**Supplementary Information:**

The online version contains supplementary material available at 10.1007/s40615-024-02276-7.

## Introduction

Racial disparities in US mortality rates have been well-documented in the last century [1]. In prior studies, age-adjusted mortality rates (AAMR) in non-Hispanic Blacks (NHB) were higher than those in non-Hispanic Whites (NHW). Geographic disparities were also described [2, 3]. Mississippi (MS) is known to have the highest cardiovascular disease (CVD) mortality rate in the US [1], the worst being in the 18-county MS Delta region, making MS the subject of major trials such as the Jackson Heart Study. The AAMR varied among counties in MS in earlier studies, and AAMRs were higher in the South and in Mississippi (MS) than in other areas [4–8].

Why do such disparities exist and persist into the twenty-first century? Intersectionality theory can help to identify the root (fundamental) causes of health disparities. Intersectionality is defined as “The inequalities produced by simultaneous and intertwined statuses and how that influences the life-course of an individual or group” [9]. The theory was put forth in 1989 by Kimberle Crenshaw in the context of feminism but has been applied to other issues such as health inequities [10, 11].

Historically, black Americans have faced deprivation, abuse, and discrimination based on multiple statuses: enslavement, foreign birth, race, ethnicity, gender, religion, lack of education, and physical appearance among others. Also termed cumulative disadvantage and multiple jeopardy, intersectionality placed NHBs at the bottom of the social hierarchy along with the indigenous population. Since colonial times, the black population had among the poorest chances for survival, longevity, and mental health. However, when considering populations’ past, it is also important to recognize that health exists along the spectrum of flourishing to death, a spectrum for which data are often sparse. With this in mind, an “inequity paradox” observed in modern public health interventions (higher-status persons benefit more from population-wide interventions) may have applied to changes such as emancipation and federal civil rights legislation, which may have benefited enslaved groups in border states like Delaware and Maryland more than those in states like Mississippi, where whole economies were based on slave labor or later sharecropping. It may be important to recognize social and geographic strata within marginalized populations leading to higher exposure to multiple risk factors for ill health in certain subpopulations [10]. Intersectionality of risks may occur in which risk factors interact in complex ways across a life-course. Socioeconomic factors are primary determinants of ill health because they determine access to resources, exposure to risk factors and access to protective factors.

A recent survey of the history of American medicine showed how it embraced and perpetuated racial inequities. As scientific methods were developed and applied in medicine, the pre-scientific beliefs persisted that differences among population groups were inherent and permanent, ordained by the Creator [12]. In the nineteenth century, leading medical journals at first perpetuated views that NHBs were inherently less susceptible to certain diseases such as tuberculosis due to their living “uncivilized” lives in nature. Likewise, journal authors portrayed NHBs as inherently prone to the vice of drunkenness. A rise in tuberculosis after the Civil War was blamed on the unsuitableness of NHBs for emancipation and their failure to evolve a level of immunity similar to NHWs [12]. Morton and others used skull measurements as evidence of white superiority [13]. This reinforced the prevailing social hierarchy that ranked people from northwest Europe above all others. As the field of genetics evolved, racial inequities were inappropriately attributed to genes. Evidence of human origins in Africa also failed to put assumptions of white supremacy to rest [14]. Counter-arguments by black leaders such as W.E.B. DuBois from the 1890s forward were ignored or dismissed [12]. As the field of genetics evolved, racial inequities were inappropriately attributed to genes [12].

Racial discrimination has been associated with negative health outcomes including mortality [15]. It is thought to account for a substantial portion of the black-white health inequity [16]. In addition to preventing access to resources, discrimination acts as a chronic stressor damaging body systems. A recent national survey documented ongoing perceived discrimination against NHBs [17].

Few studies have examined disparities by county and race/ethnicity using the latest data for the state of Mississippi. Identifying these disparities may suggest hypotheses for further research and identify populations for targeted public health interventions. This report describes racial disparities by county in MS in 1999–2020. This study also examined the hypothesis that the AAMR ratio was related to the percent of the total county population that was NHB in 1999–2020.

## Methods

Mortality data for the US and the state of Mississippi were obtained from the Centers for Disease Control and Prevention (CDC) for years 1999–2020 [18]. The data were available through CDC WONDER which has national mortality and population data produced by the Mortality Statistics Branch, Division of Vital Statistics, National Center for Health Statistics (NCHS), Centers for Disease Control and Prevention (CDC), United States Department of Health and Human Services (US DHHS). Mortality data were collected by state registries and sent to the National Vital Statistics System. Data were derived from death certificates for US residents. The number of deaths and death rates were obtained by place of residence (state and county), urbanization category, age group, race, Hispanic ethnicity, and gender at ages 35–84 years. Definitions of urbanization categories are given in the supplement table (Table S1). The age group 35–84 was chosen to focus on all adults combined, among whom chronic diseases are the main causes of mortality. An upper age limit reduces residual confounding by age with an open upper category. The CDC database uses the terms “Black or African American,” which here is shortened at times to “NHBs” for brevity, and “white.”

### Analysis

The age-adjusted mortality rate (AAMR) was calculated by county, gender—male (M) vs female (F)—and race—black (B) vs white (W)—among non-Hispanics (NH) for all causes of death. The year 2000 US population was used for age-adjustment [19]. CDC has chosen the year 2000 US populations as the default year for age-adjustment, which accounts for potential shifts in demographic distributions in the state and counties over time and for differences among counties and racial groups.

The 95% confidence intervals (CI) were shown in parentheses. The 2013 CDC classification of urbanization was used. The ratio of AAMR in NHB to NHW was computed by county. A simple linear regression model was fit with county AAMR ratio as the dependent variable and county percent NHB as the independent variable for MS counties (*n* = 82) for each gender-race group. The y-intercept, slope, and R-squared were reported and scatterplots were shown (Table S2). Stata version 16.1 (Stata Corp, LLC, College Station, TX) was used.

## Results

In 2019–2020, MS had the highest AAMR per 100,000 among states in the US for age group 35–84: 1481.4 (95% CI 1468.5–1494.4). Among NHW males (NHWM), MS had the second-highest AAMR after West Virginia. Among NHW females (NHWF), MS had the third-highest AAMR after West Virginia and Kentucky. Among NHB males (NHBM), MS was tied with Michigan for the second-highest AAMR. Among NHB females (NHBF), MS had the highest AAMR.

In Mississippi in 1999–2020 combined for NH males, AAMR was greatest in micropolitan/non-metropolitan areas in NHBs, and non-core/non-metropolitan areas in NHWs (see Supplement Fig. S1). The Black/White AAMR ratio among males was highest (1.42) in the small metropolitan areas and lowest (1.18) in the large fringe metropolitan areas. The same pattern was seen in NH females (1.36 and 1.05 respectively).

In 1999–2020 combined for NHWM, counties with the highest AAMR per 100,000 were Quitman 2150 (1936–2363), followed by Tunica, Sunflower, Covington, and Attala, the first three of which are in the Delta region (Fig. S2). For NHWF, the highest were George 1356 (1289–1424), Yalobusha, Quitman, Leflore, and Marshall counties. For NHBM, the highest were Bolivar 2853 (2729–2977), followed by Webster, Grenada, Coahoma, and Washington counties, the first and last two of which are in the Delta region. For NHBF, the highest were Bolivar 1614 (1541–1686), followed by Wilkinson, Sunflower, Coahoma, and Grenada counties.

In 1999–2020 combined for NH males, the counties with the highest ratio of AAMR in NHBs to AAMR in NHWs were Oktibbeha (1.8), Jefferson (1.7), Noxubee (1.6), Bolivar (1.6), and Sharkey (1.5) (see Supplement Fig. 2). Counties with the lowest ratio were Greene (0.9), Pontotoc (0.9), Issaquena (1.0), DeSoto (1.0), and Itawamba (1.0). Tables 1 and 2 show the rates and B/W rate ratios by county. For NH females, the counties with the highest ratio of AAMR in NHBs to AAMR in NHWs were Issaquena (2.2), Jefferson (2.0), Noxubee (1.7), Franklin (1.6), and Oktibbeha (1.6). Counties with the lowest ratio were Yalobusha (0.9), Pontotoc (0.9), Marshall (0.9), Benton (0.9), and DeSoto (1.0). As shown in Tables 1 and 2, the counties in the top 10 for both Male and Female were Oktibbeha, Jefferson, Noxubee, and Bolivar. The counties in the bottom 10 for both Male and Female were Simpson, Benton, Tishomingo, DeSoto, and Pontotoc. Interestingly, Issaquena had a low male mortality (bottom 10 for males) but a high female mortality (top 10 for females).

**Table 1:** Black/White (B/W) ratio of age-adjusted mortality rates in non-Hispanic males aged 35–84, Mississippi, 1999–2020

County	B/W ratio	County	B/W ratio	County	B/W ratio	County	B/W ratio	County	B/W ratio
Oktibbeha County, MS	1.83	Yazoo County, MS	1.42	Wayne County, MS	1.32	Hancock County, MS	1.23	Leake County, MS	1.12
Jefferson County, MS	1.73	Wilkinson County, MS	1.42	Neshoba County, MS	1.32	Union County, MS	1.23	Prentiss County, MS	1.12
Noxubee County, MS	1.61	Winston County, MS	1.41	Monroe County, MS	1.32	Marion County, MS	1.22	Tunica County, MS	1.12
Bolivar County, MS	1.58	Choctaw County, MS	1.40	Lauderdale County, MS	1.31	Calhoun County, MS	1.22	George County, MS	1.08
Sharkey County, MS	1.53	Lafayette County, MS	1.40	Holmes County, MS	1.31	Tate County, MS	1.22	Simpson County, MS	1.08
Coahoma County, MS	1.52	Adams County, MS	1.39	Panola County, MS	1.31	Perry County, MS	1.21	Attala County, MS	1.07
Smith County, MS	1.51	Webster County, MS	1.39	Lee County, MS	1.31	Jasper County, MS	1.21	Benton County, MS	1.07
Madison County, MS	1.48	Alcorn County, MS	1.38	Tallahatchie County, MS	1.31	Jackson County, MS	1.21	Tishomingo County, MS	1.05
Humphreys County, MS	1.46	Leflore County, MS	1.38	Hinds County, MS	1.30	Tippah County, MS	1.21	Covington County, MS	1.05
Warren County, MS	1.46	Jefferson Davis County, MS	1.37	Amite County, MS	1.29	Chickasaw County, MS	1.20	Itawamba County, MS	1.03
Claiborne County, MS	1.45	Montgomery County, MS	1.37	Rankin County, MS	1.29	Sunflower County, MS	1.20	DeSoto County, MS	1.02
Grenada County, MS	1.44	Carroll County, MS	1.35	Lawrence County, MS	1.29	Lamar County, MS	1.19	Issaquena County, MS	1.00
Clay County, MS	1.44	Washington County, MS	1.35	Walthall County, MS	1.28	Harrison County, MS	1.18	Pontotoc County, MS	0.92
Lowndes County, MS	1.43	Newton County, MS	1.35	Pearl River County, MS	1.27	Marshall County, MS	1.17	Greene County, MS	0.91
Lincoln County, MS	1.43	Pike County, MS	1.34	Scott County, MS	1.24	Yalobusha County, MS	1.16		
Clarke County, MS	1.43	Copiah County, MS	1.34	Stone County, MS	1.23	Kemper County, MS	1.14		
Forrest County, MS	1.43	Franklin County, MS	1.33	Jones County, MS	1.23	Quitman County, MS	1.13

**Table 2:** Black/White (B/W) ratio of age-adjusted mortality rates in non-Hispanic females aged 35–84, Mississippi, 1999–2020

County	B/W ratio	County	B/W ratio	County	B/W ratio	County	B/W ratio	County	B/W ratio
Issaquena County, MS	2.20	Newton County, MS	1.34	Walthall County, MS	1.27	Harrison County, MS	1.20	Wayne County, MS	1.13
Jefferson County, MS	2.04	Jefferson Davis County, MS	1.34	Claiborne County, MS	1.27	Leake County, MS	1.19	George County, MS	1.10
Noxubee County, MS	1.73	Forrest County, MS	1.34	Lamar County, MS	1.27	Smith County, MS	1.19	Panola County, MS	1.09
Franklin County, MS	1.60	Carroll County, MS	1.34	Montgomery County, MS	1.26	Leflore County, MS	1.19	Perry County, MS	1.08
Oktibbeha County, MS	1.58	Yazoo County, MS	1.33	Choctaw County, MS	1.26	Marion County, MS	1.19	Greene County, MS	1.06
Wilkinson County, MS	1.46	Neshoba County, MS	1.33	Washington County, MS	1.25	Jasper County, MS	1.18	Clarke County, MS	1.05
Lincoln County, MS	1.46	Pike County, MS	1.32	Warren County, MS	1.24	Covington County, MS	1.18	Tishomingo County, MS	1.05
Bolivar County, MS	1.43	Lowndes County, MS	1.31	Webster County, MS	1.23	Lee County, MS	1.18	Tate County, MS	1.04
Lafayette County, MS	1.41	Tallahatchie County, MS	1.31	Rankin County, MS	1.23	Tippah County, MS	1.18	Simpson County, MS	1.03
Winston County, MS	1.40	Copiah County, MS	1.30	Scott County, MS	1.23	Monroe County, MS	1.18	DeSoto County, MS	1.01
Sunflower County, MS	1.40	Madison County, MS	1.30	Alcorn County, MS	1.23	Itawamba County, MS	1.18	Benton County, MS	0.94
Coahoma County, MS	1.39	Humphreys County, MS	1.30	Amite County, MS	1.22	Jones County, MS	1.17	Marshall County, MS	0.92
Sharkey County, MS	1.37	Attala County, MS	1.28	Holmes County, MS	1.22	Union County, MS	1.17	Pontotoc County, MS	0.89
Grenada County, MS	1.35	Clay County, MS	1.28	Lauderdale County, MS	1.22	Stone County, MS	1.16	Yalobusha County, MS	0.87
Hinds County, MS	1.35	Pearl River County, MS	1.28	Chickasaw County, MS	1.21	Hancock County, MS	1.14		
Adams County, MS	1.34	Calhoun County, MS	1.28	Prentiss County, MS	1.21	Lawrence County, MS	1.14		
Kemper County, MS	1.34	Tunica County, MS	1.27	Jackson County, MS	1.20	Quitman County, MS	1.14		

**Fig. 1:**
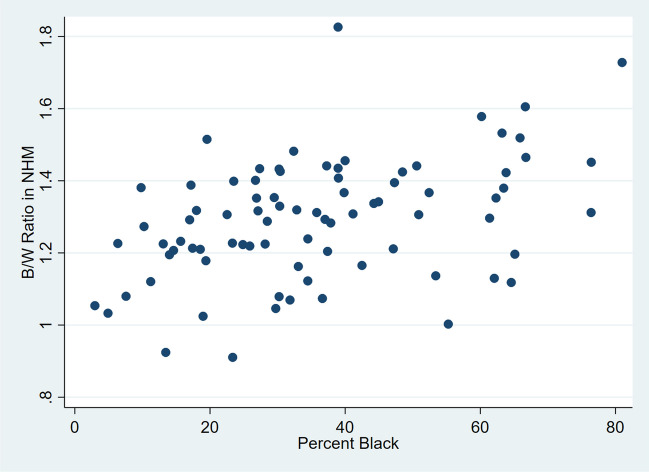
Scatterplot of county B/W AAMR ratio in non-Hispanic males by county percent black

Next, the hypothesis that the AAMR ratio was related to the percent of the total county population that was NHB in 1999–2020 was examined. In NH men, the percentage ranged from 3.0% in Tishomingo County to 81.0% in Jefferson County. In NH men the AAMR ratio was correlated with the percent NHB: Spearman R = 0.41 (*p* = 0.0001). In a linear regression model with AAMR ratio as the dependent variable and % NHB as the independent variable, the slope was 0.004 (*p* < 0.0001; 95% CI 0.002–0.006; R-squared = 0.18) and the y-intercept was 1.16. Figure 1 shows a scatter plot for NHM.

**Fig. 2:**
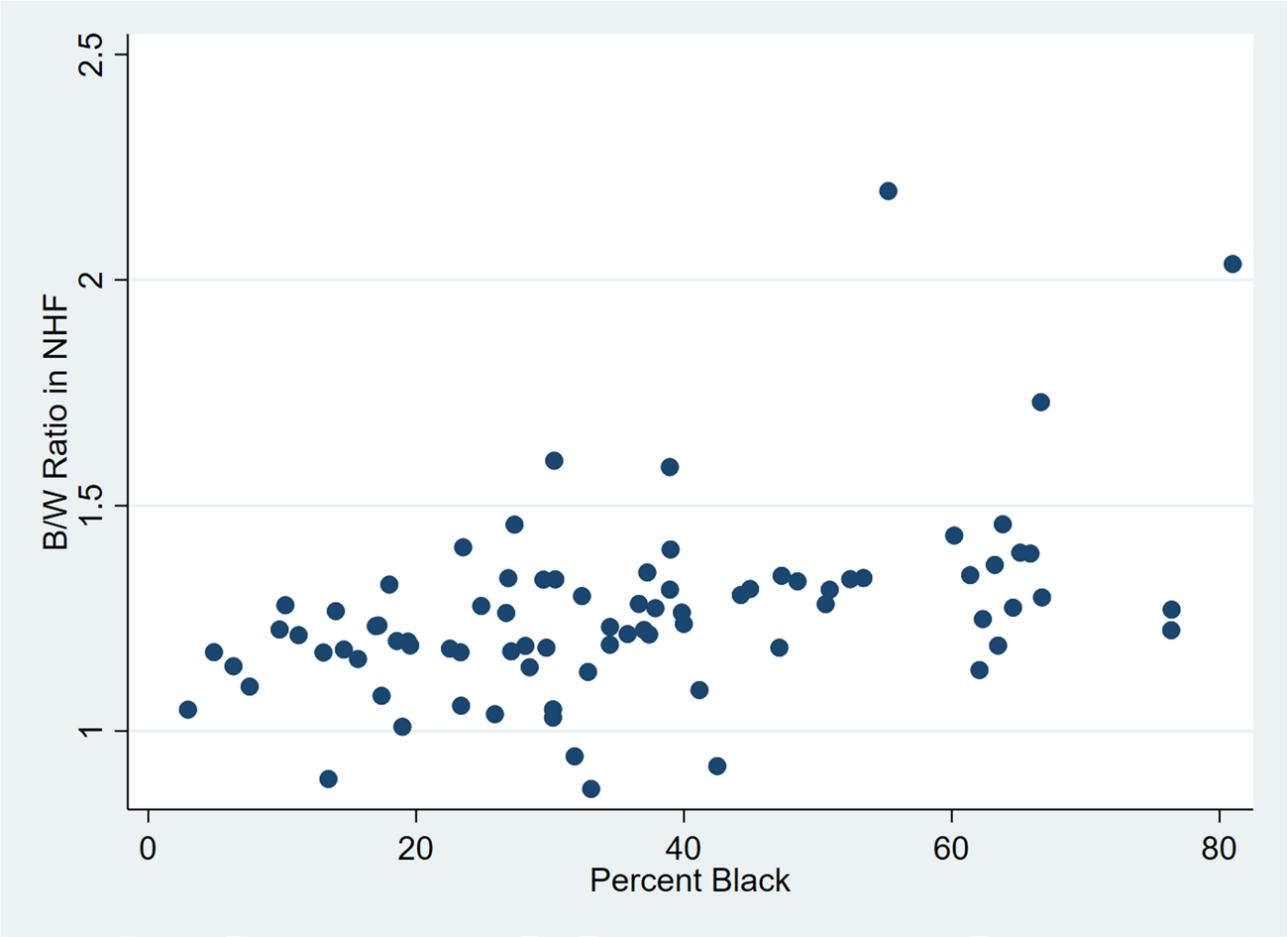
Scatterplot of county B/W AAMR ratio in non-Hispanic females by county percent black

In NH women, the AAMR ratio was correlated with the percent NHB: Spearman R = 0.50 (*p* < 0.0001). In a linear regression model with AAMR ratio as the dependent variable and % NHB as the independent variable, the slope was 0.005 (*p* < 0.0001; 95% CI 0.003–0.007; R-squared = 0.21) and the y-intercept was 1.08. Figure 2 shows a scatter plot for NHF.

## Discussion

This study documented major geographic and racial disparities in mortality from all causes within MS for age group 35–84 years. High AAMR was concentrated in the delta region for NHBM and NHBF. Four of the five top counties for each were in the MS Delta region where CVD mortality was higher. Racial disparities also varied greatly by county. In 1999–2020 for NH males, the ratio of AAMR in NHBs to NHWs varied from 0.9 to 1.8. In NH females, the ratio varied from 0.9 to 2.2. In most counties, AAMR was substantially higher in NHBs. In both genders, the ratio was significantly correlated with the percent of the population that was NH Black. These findings may be useful to researchers and health planners in guiding their efforts to understand and alleviate health disparities.

### Previous Studies

In the past half-century, US agencies have created atlases of US mortality mapping age-adjusted death rates by county [2]. Formanack et. al used a modeling approach to examine the associations of race (proportion white), class (socioeconomic status), and place (county) with the risk of cause-specific death in the US [20]. For 12 leading causes of death, Bayesian regression models were fit. A higher proportion of white residents was associated with lower death rates for 8 of the 12 causes. An advantaged cluster of counties based on area deprivation index (ADI) was seen for 9 of 12 causes. The years which were examined were 1999–2019 and for COVID-19, 2020–2021. Race, class, and place contributed to mortality, but contributions differed by cause of death.

Examination of MS on atlas maps shows an important within-state variation [2]. A study of county-level US mortality in 1980–2014 showed substantial variation in rates of death from neoplasms and cardiovascular disease among MS counties. However, analyses by race/ethnicity were not presented. This research corrected national data for “garbage codes” in 1980–2014 [4].

In 2017, CVD mortality was higher in MS than in any other state [21]. Race-sex disparities in MS as well as mortality trends were previously reported. At ages 35 and older in 2000–2018, age-adjusted CVD death rates were higher in men than women and declined by a third, more rapidly in 2000–2009 than in 2010–2018. Age-adjusted CVD death rates were higher in NHBs than in NHWs and declined more rapidly in 2000–2012 than in 2013–2018; however, relative rates of decline were similar in the two groups. Death rates were lowest in NH white women and highest in NH black men. Unfortunately, no analyses by county or region were presented. For example, no data were presented for the 18 counties in the MS delta region, said to be the focus of special programs by the MS State Health Department. Trends in heart disease mortality at ages 25 and older in MS declined between 1980 and 2013 [6]. The average annual change was − 1.34% in NHBs and − 1.60% in NHBs. Coronary heart disease death rates declined while heart failure death rates increased [7]. In 2000–2016, MS stroke death rates declined before but not after 2009 [8]. NHBs had the smallest decline. From 2000 to 2018, cardiovascular disease death rates in MS declined similarly for NHBs and NHWs [21]. Studies of heart disease in 1980–2013 and stroke in 2000–2016 reported several similar disparities and trends [6, 8]. In contrast to the former reports, increasing hypertension mortality in 2000–2018 was reported [5]. Similarly, rates of death coded to heart failure increased from 1980–2013 [7]. An earlier report documented increasing CVD mortality rates between 1984 and 1995 in NHBs in MS [22].

As reviewed above, intersectionality theory is useful both in explaining observed inequities in mortality among races and areas of Mississippi in recent times. Although this study was limited to mortality data, previous studies cited above suggest that multiple statuses of NHBs in Mississippi, especially the Delta counties, may render them subject to discrimination and other factors contributing to lower longevity: race, ethnicity, gender, religion, lack of education, poverty, neighborhood, and physical appearance, among others. Both institutional and interpersonal racism may negatively affect survival. In 2017, a nationally representative survey of US adults asked if they ever experienced discrimination based on race in both interpersonal and institutional domains [17]. Compared to NHWs, NHBs were significantly more likely to report discrimination in all domains. Regarding health, 32% of NHBs had experienced discrimination going to a doctor or clinic, and 22% of NHBs had avoided seeking care because of anticipated discrimination. After adjustments for multiple confounders, NHBs had nearly seven times the odds as NHWs of reporting these forms of discrimination. An analysis of data collected in 2017–2022 from over 40,000 individuals found that 49% of NHBs report at least one of seven types of discrimination in a health care setting [23]. An additive interaction was found between black race and less education. Future research should combine mortality data with other data to identify social factors related to racial inequities in mortality. A study of the Social Vulnerability Index found the socioeconomic factor was most closely related to mortality among Mississippi counties [24]. A discussion of how intersectionality theory could inform public health interventions or policies targeted at reducing health disparities has been published [10]. Thus, the present study is consistent with previous studies in showing substantial variations in AAMR among counties and substantially higher AAMR in NHB compared to NHW. This study also shows that although there is variation in the degree of disparity among counties, NHBs had higher AAMR than NHWs in the vast majority of counties (Figs. S3 and S4).

### Limitations

Studies analyzing national vital statistics data including mortality may be limited by inaccuracies in death registration and population enumeration and estimation. In the US, death registration is considered to be nearly complete. However, a number of population subgroups may be undercounted by the US Census, especially minority groups [25–27]. This could cause bias in death rates. Errors in the assignment of cause of death may also occur. A study of county-level US death rates in 1980–2014 examined the problem of “garbage codes” in underlying cause of death statistics [4]. However, the present analysis of mortality from all causes of death avoided this problem. Ecologic analysis is subject to bias and confounding and hence cannot provide causal inference regarding observed associations.

## Conclusions

The AAMR in MS varied greatly among counties as did the Black/White ratio of AAMR. Further research is needed to explain this geographic variation in racial disparity.

## Supplementary Information

Below is the link to the electronic supplementary material.

Supplementary file1 (DOCX 17.9 KB)

Supplementary file2 (DOCX 18 KB)

Supplementary file3 (PPTX 83 KB)

Supplementary file4 (PPTX 143 KB)

Supplementary file5 (PNG 114 KB)

Supplementary file6 (PNG 113 KB)

## Data Availability

The datasets analyzed in the current study were obtained via the publicly accessible “CDC Wonder” database (https://wonder.cdc.gov/). Data are in the public domain, provided as a service by the Centers for Disease Control and Prevention (CDC), and were used in compliance with CDC Wonder’s Data Use Agreement. The raw datasets extracted during the analysis can be sent digitally by the corresponding author upon reasonable request.
