# Supplementary material for: Geographic Variation in Racial Disparities in Age-Adjusted Mortality Rates in Mississippi
Source: J Racial Ethn Health Disparities. 2025 Jan 20;13(1):641–8. doi: 10.1007/s40615-024-02276-7 (PMC12795875; doi:10.1007/s40615-024-02276-7)
Supplement: Supplementary file 1 — Supplementary file1 (DOCX 17.9 KB) [file 40615_2024_2276_MOESM1_ESM.docx]

**SUPPLEMENT**

TABLE OF CONTENTS

Table S1. The 2013 National Center for Health Statistics urbanization classification

Table S2. Linear regression analysis with black/white ratio of age-adjusted mortality rates as dependent variable and percent of the county population that was non-Hispanic black by gender: Mississippi, 1999-2020

Figure S1. Age-adjusted rates per 100,000 for all causes of death by race and 2013 urbanization category in those aged 35-84 years by county: Mississippi, 1999-2020

Figure S2. Ratio of age-adjusted rates per 100,000 for all causes of death in black males to white males among non-Hispanics aged 35-84 years by county: Mississippi, 1999-2020

Figure S3. Age-adjusted mortality rate among non-Hispanic blacks aged 35-84 years by county, Mississippi, 1999-2020

Figure S4. Age-adjusted mortality rate among non-Hispanic whites aged 35-84 years by county, Mississippi, 1999-2020

Table S1. The 2013 National Center for Health Statistics urbanization classification

| Large central metro | Metropolitan statistical areas (MSAs) of one million or more population that have been identified by NCHS classification rules as central because they contain all or part of a principal city of the area |
| --- | --- |
| Large fringe metro | the remaining counties (similar to suburbs) in MSAs of one million or more |
| Medium metro | MSAs of 250,000 to 999,999 population |
| Small metro | MSAs with populations under 250,000 |
| Nonmetropolitan, micropolitan | Nonmetropolitan, in a micropolitan statistical area |
| Nonmetropolitan, noncore | Nonmetropolitan, not in a micropolitan statistical area |

Source: Ingram DD, Franco SJ. NCHS urban-rural classification scheme for counties.

National Center for Health Statistics. Vital Health Stat 2(154). 2012.

Table S2. Linear regression analysis with black/white ratio of age-adjusted mortality rates as dependent variable and percent of the county population that was non-Hispanic black by gender: Mississippi, 1999-2020

| Gender | Coeff | SE | t | p | R-squared |
| --- | --- | --- | --- | --- | --- |
| Male | 0.004 | 0.001 | 4.14 | 0.0001 | 0.18 |
|  |  |  |  |  |  |
| Female | 0.005 | 0.001 | 4.63 | 0.0001 | 0.21 |
|  |  |  |  |  |  |
