# Supplementary figures and images for: Geographic Variation in Racial Disparities in Age-Adjusted Mortality Rates in Mississippi
Source: J Racial Ethn Health Disparities. 2025 Jan 20;13(1):641–8. doi: 10.1007/s40615-024-02276-7 (PMC12795875; doi:10.1007/s40615-024-02276-7)

## Slide 1
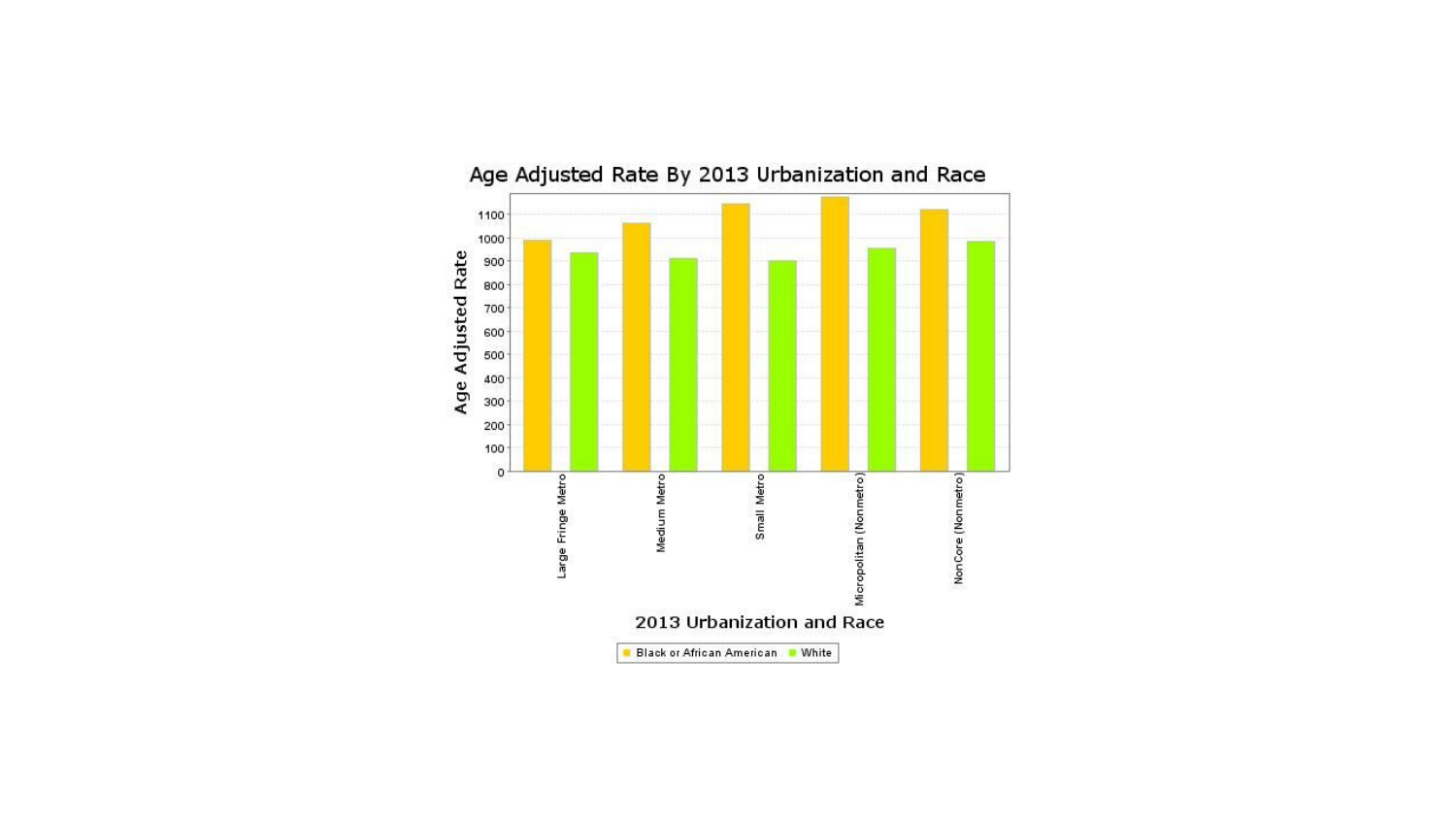

Supplement: Supplementary file 3 — Supplementary file3 (PPTX 83 KB) [file 40615_2024_2276_MOESM3_ESM.pptx]

## Slide 1
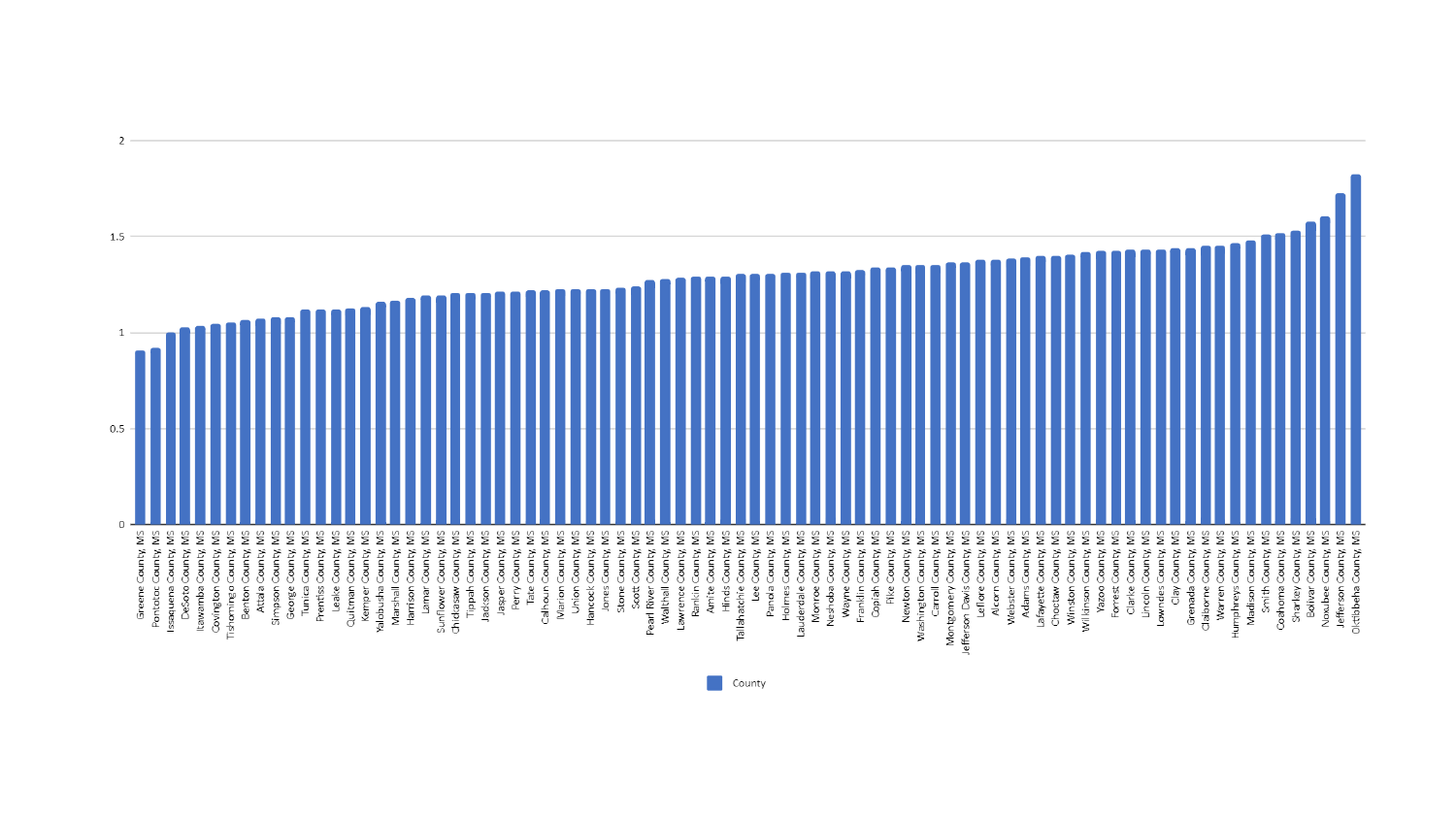

Supplement: Supplementary file 4 — Supplementary file4 (PPTX 143 KB) [file 40615_2024_2276_MOESM4_ESM.pptx]

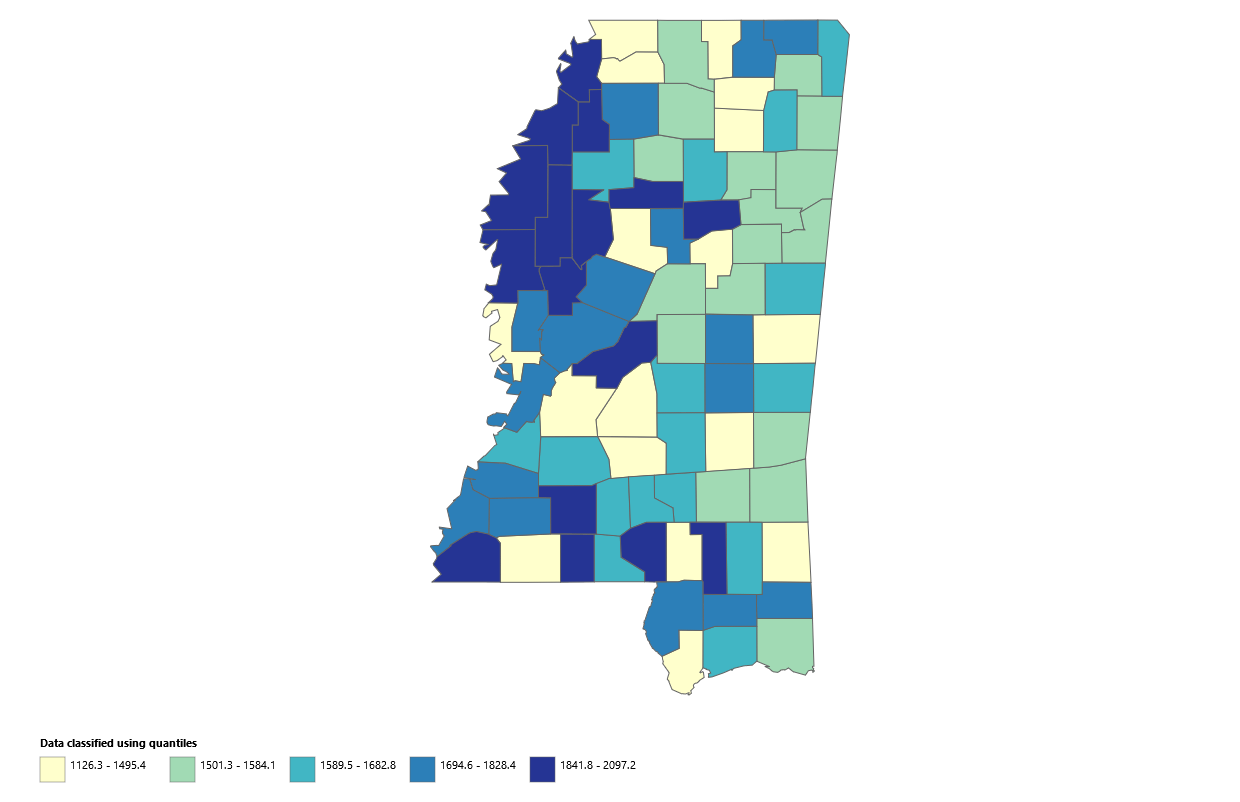

Supplement: Supplementary file 5 — Supplementary file5 (PNG 114 KB) [file 40615_2024_2276_MOESM5_ESM.png]

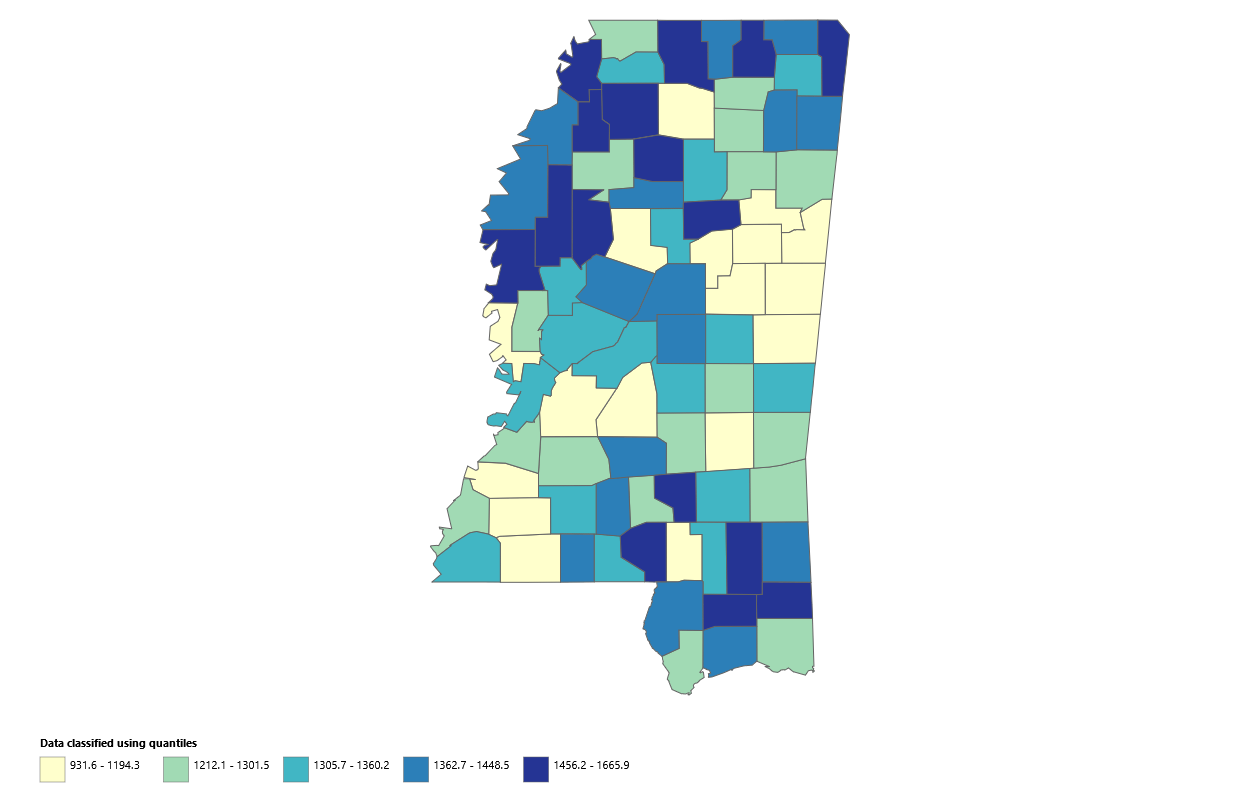

Supplement: Supplementary file 6 — Supplementary file6 (PNG 113 KB) [file 40615_2024_2276_MOESM6_ESM.png]
